# A New Operation for Vesico-Vaginal Fistula

**Published:** 1894-03

**Authors:** 


A NEW OPERATION FOR VESICO-VAGINAL FISTULA.—Trendelenburg (*Deutsche medicinische Wochenschrift*, No. 40, 1893,) describes a method of operation he has improvised for those cases of vesico-vaginal fistula in which the usual operation is impossible because of the relation of the cervix to the fistula. The patient is placed in the Trendelenburg position, and a lateral incision made above the symphysis pubis, severing the recti muscles and opening the pre-vesical space. The bladder is incised transversely, and provisionally sutured to the skin wound. He then freshens the edges of the fistula and closes it with catgut sutures. The bladder wound is closed with catgut sutures, leaving a small opening through which a drainage tube, attached to a long rubber tube, is introduced. The muscles are united to the symphysis by means of wire sutures and the wound packed with gauze. Drainage is continued about fourteen days, or until the fistula is completely healed, the patient lying on her side, preferably upon a water-bag. Because of the danger of hernia following this operation, Trendelenburg advises his patients to wear an abdominal supporter.—*Ex.*

